# A practical nomogram included hyperlipidemia for predicting lymph node metastasis in patients with superficial esophageal squamous cell carcinoma

**DOI:** 10.1097/MD.0000000000035891

**Published:** 2023-11-17

**Authors:** Jing Wang, Xiangji Liu, Tao Mao, Zitong Xu, Hanqing Li, Xiaoyu Li, Xuan Zhou, Yuning Chu, Minghan Ren, Zibin Tian

**Affiliations:** a Department of Gastroenterology, the People’s Hospital of Rizhao, Rizhao, Shandong Province, China; b Department of Gastroenterology, the Affiliated Hospital of Qingdao University, Qingdao, Shandong Province, China; c Department of Pathology, the Affiliated Hospital of Qingdao University, Qingdao, Shandong Province, China; d Department of Nutriology, the Affiliated Hospital of Qingdao University, Qingdao, Shandong Province, China.

**Keywords:** endoscopic resection, hypertriglyceridemia, lymph node metastasis, nomogram, superficial esophageal squamous cell carcinoma

## Abstract

To select an optimal treatment, it is crucial to evaluate the risk of lymph node metastasis (LNM) in patients with superficial esophageal squamous cell carcinoma (SESCC). The research aimed to explore more risk factors than before and construct a practical nomogram to predict LNM in patients with SESCC. We retrospectively reviewed 1080 patients diagnosed with esophageal cancer who underwent esophagectomy with lymphadenectomy between January 2013 and October 2021 at the Affiliated Hospital of Qingdao University. The clinical parameters, endoscopic features, and pathological characteristics of the 123 patients that were finally enrolled in this study were collected. The independent risk factors for LNM were determined using univariate and multivariate analyses. Using these factors, a nomogram was constructed to predict LNM. LNM was observed in 21 patients. Univariate analysis showed that the absence or presence of hypertriglyceridemia, tumor location, lesion size, macroscopic type, invasion depth, differentiation, absence or presence of lymphovascular invasion (LVI), and perineural invasion were significantly associated with LNM. According to the multivariate analysis, hypertriglyceridemia, tumors located in the lower thoracic esophagus, lesion size > 20 mm, submucosal invasion, and LVI were independent risk factors for LNM. A nomogram was established using these 5 factors. It showed good calibration and discrimination. Hypertriglyceridemia, tumors located in the lower thoracic esophagus, lesion size > 20 mm, submucosal invasion, and LVI were independent risk factors for LNM. A nomogram was constructed using these 5 factors. This model can help clinicians assess the risk of LNM in patients with SESCC for optimal treatment selection.

## 1. Introduction

Esophageal cancer is one of the most common malignant tumors. Its incidence ranks seventh and mortality ranks sixth overall.^[1]^ Superficial esophageal squamous cell carcinoma (SESCC) refers to squamous cell carcinoma that is limited to the mucosa (T1a) and submucosa (T1b), regardless of the presence or absence of lymph node metastasis (LNM).^[2]^ With the development of endoscopic techniques and improvement in human health awareness, SESCC has been increasingly detected. Patients with SESCC generally have a better prognosis than those with advanced esophageal cancer. Several studies have reported that the 5-year survival rate is higher than 90.0% for pT1a and above 70.0% for pT1b.^[3–5]^

Radical esophagectomy with lymph node dissection is the standard treatment for SESCCs. Radical surgery is associated with considerable morbidity and mortality.^[6–8]^ Moreover, it is not suitable to perform radical surgery in patients with advanced age or in poor general condition.^[9]^ Endoscopic resection (ER) is a minimally invasive treatment that can maintain the integrity of the esophagus and ensure the quality of life of patients, and it has gradually become an alternative to radical surgery.^[10]^ The Japan Esophageal Society (JES) proposed that the absolute indications for ER include lesions confined within the epithelium (EP) and lamina propria mucosa (LPM). The relative indications include lesions extending up to the muscularis mucosa (MM) or slightly infiltrating the submucosa (no more than 200 μm); however, these are associated with an elevated risk of LNM.^[11]^ Considering that lymphadenectomy cannot be performed as part of ER, if patients with SESCC have positive LNM, ER is no longer considered an option. Therefore, it is critical to assess the risk of LNM in order to choose the optimal treatment.

Based on existing preoperative examination methods, such as computed tomography, magnetic resonance imaging, and endoscopic ultrasonography, it is still difficult to accurately detect the presence of LNM.^[12–14]^ In addition, the clinicopathological risk factors associated with LNM in SESC remain unclear. Therefore, this study aimed to determine the risk factors for LNM in patients with SESCC and establish a practical nomogram based on these factors to guide the treatment of patients with SESCC.

## 2. Materials and methods

### 2.1. Patients

**Figure 1.**
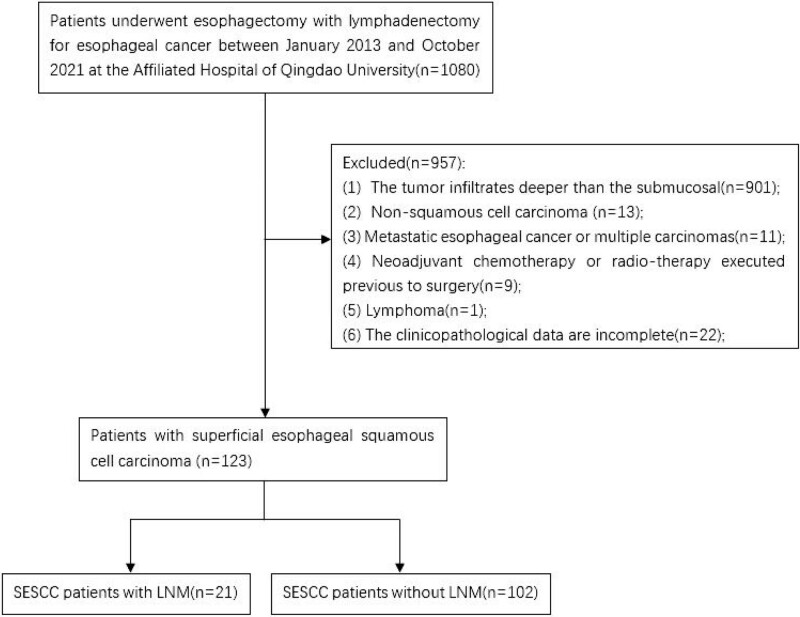
Flowchart of patients included in the study.

We retrospectively reviewed all patients with histopathologically-confirmed esophageal cancer who underwent esophagectomy with lymphadenectomy between January 2013 and October 2021 at the Affiliated Hospital of Qingdao University. A total of 1080 patients were reviewed. The exclusion criteria were: tumor infiltrates deeper than the submucosal, non-squamous cell carcinoma, metastatic esophageal cancer or multiple carcinomas, neoadjuvant chemotherapy or radiotherapy executed prior to surgery, lymphoma, incomplete clinicopathological data, and other life-threatening diseases. Finally, 123 patients with SESCC were included in this study (Fig. 1).

The use of medical record data for this study was approved by the Ethical Committee of the Affiliated Hospital of Qingdao University, China (QYFYWZLL27183). The retrospective study protocol complied with all provisions of the Helsinki Declaration. Informed consent was obtained from each participant.

### 2.2. Data collection

**Figure 2.**
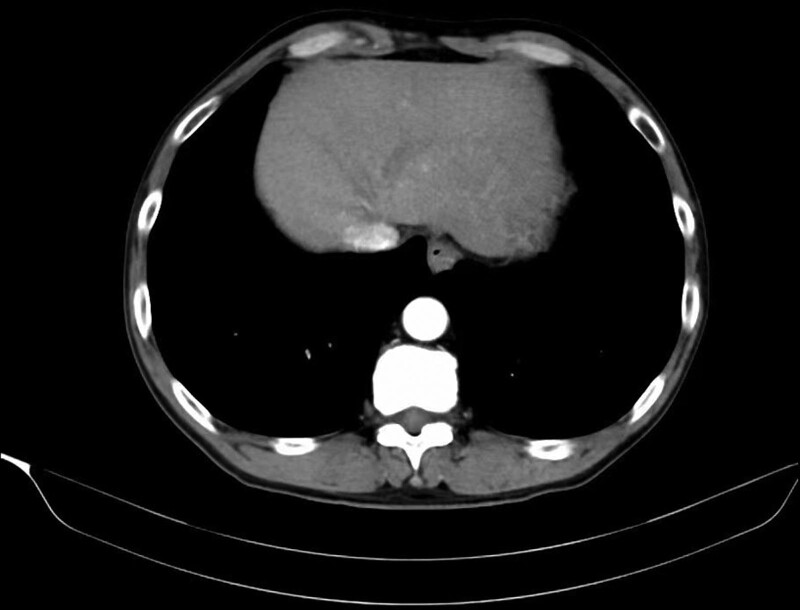
The imageological manifestations of SESCC. SESCC = superficial esophageal squamous cell carcinoma.

**Figure 3.**
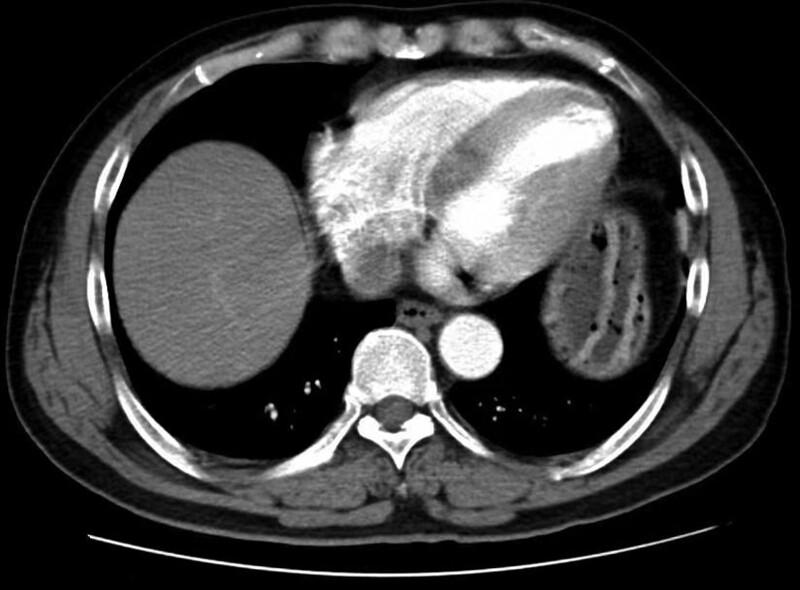
The imageological manifestations of SESCC. SESCC = superficial esophageal squamous cell carcinoma.

**Figure 4.**
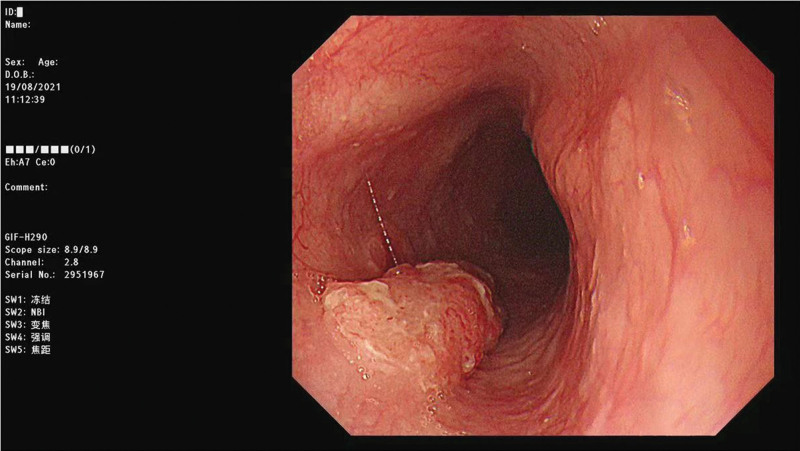
The endoscopic manifestations of SESCC. SESCC = superficial esophageal squamous cell carcinoma.

**Figure 5.**
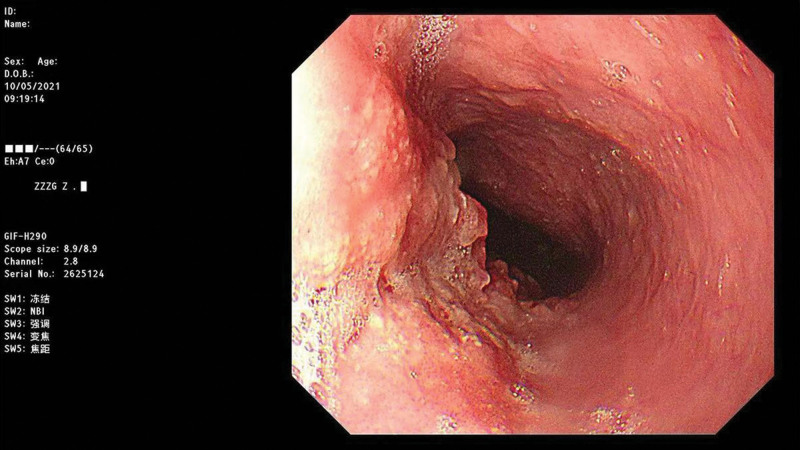
The endoscopic manifestations of SESCC. SESCC = superficial esophageal squamous cell carcinoma.

**Figure 6.**
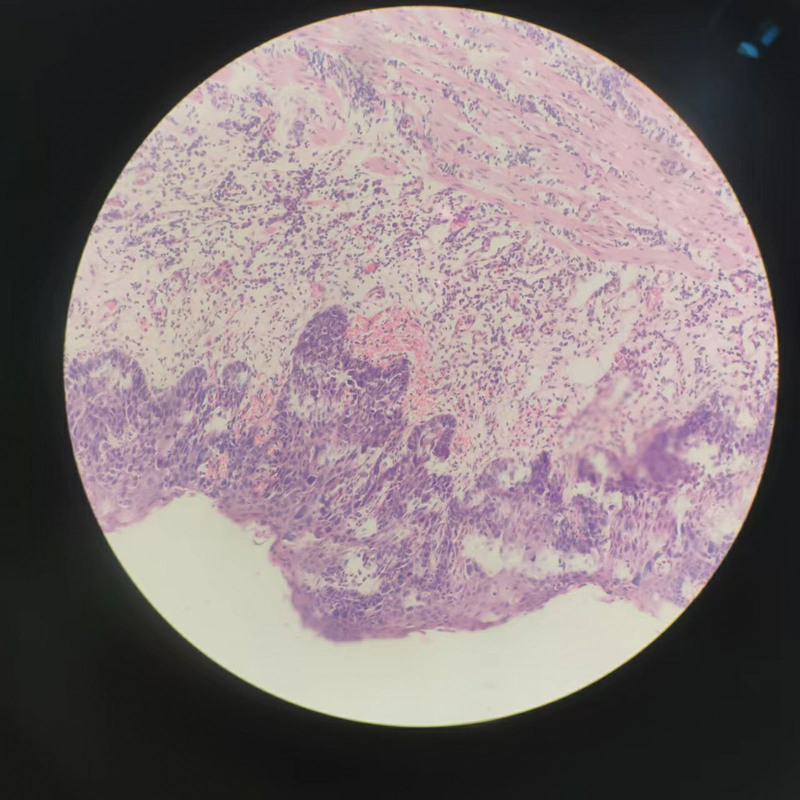
The pathological manifestations of SESCC. Figure 6 shows tumor invasion into the lamina propria mucosa. SESCC = superficial esophageal squamous cell carcinoma.

**Figure 7.**
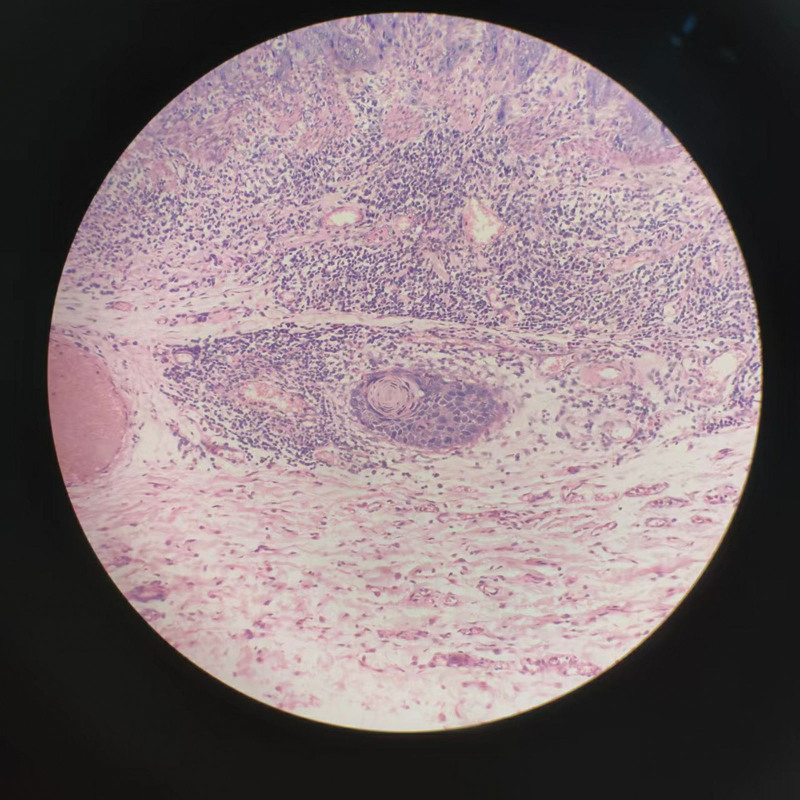
The pathological manifestations of SESCC. Figure 7 shows tumor invasion into the submucosa. SESCC = superficial esophageal squamous cell carcinoma.

All the included patients underwent esophagectomy with lymphadenectomy. Surgical specimens were fixed with formaldehyde and cut into 3-mm-thick slices. The histopathological examinations were independently performed by 2 experienced pathologists. LNM was defined as the presence of cancerous tissue within the lymph node capsule. We collected the clinical, endoscopic, and pathological characteristics of all enrolled patients in this study, including age, sex, body mass index (BMI), presence of hypertension, heart disease, diabetes mellitus, hypercholesterolemia, hypertriglyceridemia, smoking and drinking history, family history of cancer, family history of gastrointestinal cancer, tumor location, lesion size, number of tumors, macroscopic type, invasion depth, differentiation, presence of lymphovascular invasion (LVI), perineural invasion, and LNM. Figures 2–7 shows the imageological, endoscopic and pathological manifestations of SESCC.

The definitions of hypercholesterolemia and hypertriglyceridemia were serum levels of total cholesterol ≥ 220 mg/dL and total triglyceride (TG) ≥ 150 mg/dL, respectively.^[15]^ The location of the primary tumor was defined by the location of the upper end of the tumor in the esophagus, according to the American Joint Committee on Cancer/Union for International Cancer Control 8th edition TNM classification of esophageal cancer.^[16]^ Tumor locations were classified as upper-middle thoracic esophagus (<30 cm from the incisors) and lower thoracic esophagus (≥30 cm from the incisors). The macroscopic types were divided into the following 3 types: I (protruded), II (flat), and III (depressed). Tumor size was graded as ≤ 20 mm or > 20 mm for further analysis. In line with the Japanese Classification of Esophageal Carcinoma,^[17]^ tumors were classified into the following 4 groups based on invasion depth: mucosa, upper third of the submucosa (SM1), and middle third of the submucosa (SM2) + lower third of the submucosa (SM3). For differentiation, all tumors were divided into well, moderately, and poorly differentiated tumors. When there were multiple tumors, we selected the one with the deepest infiltration as the research sample. However, when the invasion depth was identical, we chose the largest lesion.^[17]^

### 2.3. Statistical analysis

Continuous variables were converted to categorical variables. Since the mean age of the patients was 59.6 years, we divided the patients into 2 groups using 60 years as a cutoff value. According to obesity criteria, the included patients were classified into 2 groups: non-obese (BMI < 28 kg/m^2^) and obese (BMI ≥ 28 kg/m^2^). The χ^2^ test or Fisher exact test was used to examine the differences between LNM and categorical variables. Multivariate logistic regression analysis was performed on factors that were significant in the univariate analysis to identify the independent risk factors for LNM in patients with SESCC. Linear analysis was used to rule out multicollinearity. R software (version 4.1.3) was used to construct a nomogram based on independent risk factors. The area under the receiver operating characteristic curve and concordance index were calculated to evaluate the discrimination of the nomogram. A calibration plot, Brier score, and Hosmer-Lemeshow goodness-of-fit tests were used to assess the calibration of the nomogram. To reduce overfitting bias, the nomogram was subjected to 1000 bootstrap resamples for internal validation. *P* < .05 (two-sided) indicates the difference is statistically significance. Statistical analyses were performed via IBM SPSS Statistics for Windows version 26.0 (IBM Corp., Armonk, NY) and R, version 4.1.3 (R Foundation for Statistical Computing).

## 3. Results

### 3.1. Clinical characteristics

We assessed 1080 patients who were diagnosed with esophageal cancer and underwent esophagectomy with lymphadenectomy. After applying the selection criteria, 123 SESCC patients were included in this study. Based on the pathologic examinations, 21 (17.1%) patients developed LNM while 102 (82.9%) patients did not. The mean age of patients was 59.6 (range, 44–79) years. The majority of patients with SESCC were men (89.45%), with only 10.6% women. The clinical characteristics of the patients with SESCC are listed in Table 1. The Univariate analysis revealed that the absence or presence of hypertriglyceridemia was significantly correlated with LNM (*P* = .006). SESCC patients with hypertriglyceridemia had a higher risk of developing LNM. However, age, sex, BMI, presence of hypertension, heart disease, diabetes mellitus, and hypercholesterolemia, smoking and drinking history, family history of cancer, family history of gastrointestinal cancer were not significantly associated with LNM.

**Table 1:** Lymph node metastasis risk according to clinical parameters.

	Total (n = 123), n%	LMN negative (n = 102), n%	LMN positive (n = 21) n%	*P* value
Age (yr)				.717
≤ 60	63 (51.2)	53 (52.0)	10 (47.6)	
> 60	60 (48.8)	49 (48.0)	11 (52.4)	
Sex				.575
Male	110 (89.4)	90 (88.2)	20 (95.2)	
Female	13 (10.6)	12 (11.8)	1 (4.8)	
BMI				.27
< 28	115 (93.5)	97 (95.1)	18 (85.7)	
≥28	8 (6.5)	5 (4.9)	3 (14.3)	
Hypertension				.12
Absence	95 (77.2)	82 (80.4)	13 (61.9)	
Presence	28 (22.8)	20 (19.6)	8 (38.1)	
Heart disease				.487
Absence	113 (91.9)	95 (93.1)	18 (85.7)	
Presence	10 (8.1)	7 (6.9)	3 (14.3)	
Diabetes mellitus				.973
Absence	114 (92.7)	94 (92.2)	20 (95.2)	
Presence	9 (7.3)	8 (7.8)	1 (4.8)	
Hypercholesterolemia				.99
Absence	88 (71.5)	73 (71.6)	15 (71.4)	
Presence	35 (28.5)	29 (28.4)	6 (28.6)	
Hypertriglyceridemia				**.006**
Absence	93 (75.6)	82 (80.4)	11 (52.4)	
Presence	30 (24.4)	20 (19.6)	10 (47.6)	
Drinking				.554
Absence	42 (34.1)	36 (35.3)	6 (28.6)	
Presence	81 (65.9)	66 (64.7)	15 (71.4)	
Smoking				.334
Absence	34 (27.6)	30 (29.4)	4 (19.0)	
Presence	89 (72.4)	72 (70.6)	17 (81.0)	
Family history of cancer				.346
Absence	92 (74.8)	78 (76.5)	14 (66.7)	
Presence	31 (25.2)	24 (23.5)	7 (33.3)	
Family history of gastrointestinal cancer				.334
Absence	100 (81.3)	85 (83.3)	15 (71.4)	
Presence	23 (18.7)	17 (16.7)	6 (28.6)	

BMI *=* Body mass index, LNM *=* Lymph node metastasis.

### 3.2. Analysis of endoscopic features

Data on the endoscopic features are shown in Table 2. Univariate analysis showed that there were significant correlation between tumor location, lesion size, macroscopic type and LNM (*P* = .001, *P* < .001, *P* = .016, respectively). Tumors were more likely to occur in the lower thoracic esophagus (n = 66,53.7%), most of which were single (n = 108, 87.8%). Lesions > 20 mm (n = 50, 40.7%) were more prone to developing LNM. According to the macroscopic types, there were 67 cases of I (protruded) type, accounting for 54.5%, while only 17 of III (depressed) type, accounting for 13.8% occurred. The number of tumors did not reach statistical significance level (*P* = .492).

**Table 2:** Lymph node metastasis risk according to endoscopic features.

	Total (n = 123), n%	LMN negative (n = 102), n%	LMN positive (n = 21) n%	*P* value
Location				**.001**
Upper + Middle	57 (46.3)	54 (52.9)	3 (14.3)	
Lower	66 (53.7)	48 (47.1)	18 (85.7)	
Lesion size				**<.001**
≤20mm	73 (59.3)	68 (66.7)	5 (23.8)	
>20mm	50 (40.7)	34 (33.3)	16 (76.2)	
Number of tumors				.492
Single	108 (87.8)	91 (89.2)	17 (81.0)	
Multitude	15 (12.2)	11 (10.8)	4 (19.0)	
Macroscopic type				**.025**
I(Protruded)	67 (54.5)	53 (52.0)	14 (66.7)	
II(Flat)	39 (31.7)	37 (36.3)	2 (9.5)	
III(Depressed)	17 (13.8)	5 (23.8%)	12 (11.8)	

LNM = lymph node metastasis.

### 3.3. Analysis of the pathological characteristics

Table 3 summarizes the pathological characteristics of the enrolled patients. Among the 123 patients with SESCC, 21 had LNM while 102 did not. The incidence of LNM in SESCC patients was 17.1%. The LNM rates were 2.0%, 8.3%, and 45.9% for tumors confined to the mucosa, SM1, and SM2 + SM3, respectively. To verify whether the JES guidelines for endoscopic treatment of SESCC were applicable to Chinese patients, we performed further analysis to classify the depth of infiltration into EP + LPM, MM + SM1, and SM2 + SM3. Lesions confined within the EP and LPM conformed to the absolute indication, and no LNM was observed in these patients (Table 4). Patients with tumors infiltrating the MM and SM1 showed consistency with the relative indications, and LNM occurred in 6.2% of them. However, tumors that infiltrated SM2 and SM3 were related to a higher risk of LNM (*P* < .001). From this point of view, the guidelines are applicable to Chinese patients.

**Table 3:** Lymph node metastasis risk according to pathological characteristics.

	Total (n = 123), n%	LMN negative (n = 102), n%	LMN positive (n = 21) n%	*P* value
Invasion depth				**<.001**
Mucosa	50 (40.7)	49 (48.0)	1 (4.8)	
SM1	36 (29.3)	33 (32.4)	3 (14.3)	
SM2 + SM3	37 (30.1)	20 (19.6)	17 (81.0)	
Differentiation				**.004**
Well differentiated	29 (23.6)	28 (27.5)	1 (4.8)	
Moderately differentiated	67 (54.5)	57 (55.9)	10 (47.6)	
Poorly differentiated	27 (22.0)	17 (16.7)	10 (47.6)	
LVI				**<.001**
Absence	108 (87.8)	97 (95.1)	11 (52.4)	
Presence	15 (12.2)	5 (4.9)	10 (47.6)	
Perineural invasion				**.001**
Absence	116 (94.3)	100 (98.0)	16 (76.2)	
Presence	7 (5.7)	2 (2.0)	5 (23.8)	

LNM *=* Lymph node metastasis, LVI *=* Lymphovascular invasion, SM1 *=* upper third of submucosa layer, SM2 *=* middle third of submucosa layer, SM3 *=* lower third of submucosa layer.

**Table 4:** Lymph node metastasis in superficial esophageal cancer according to the invasion depth.

	LNM (%)	*P* value
EP + LPM	0/21 (0.0)	**<.001**
MM + SM1	4/65 (6.2)	
SM2 + SM3	17/37 (45.9)	

EP *=* epithelium, LNM *=* Lymph node metastasis, LPM *=* lamina propria mucosa, MM *=* muscularis mucosa, SM1 *=* upper third of submucosa layer, SM2 *=* middle third of submucosa layer, SM3 *=* lower third of submucosa layer.

Regarding differentiation, moderately differentiated type (n = 67, 54.5%) formed the most of SESCC. The proportion of poorly differentiated type (n = 27,22%) was the lowest, but they had the highest incidence of LNM among the 3 types. LVI and perineural invasion were observed in 12.2% and 5.7% of the study population, respectively. In terms of LVI, only 15 patients developed LVI, of whom 10 had LNM. Similarly, only 7 patients had perineural invasion, but 5 of them had LNM. These 2 factors were closely associated with the absence or presence of LNM.

### 3.4. Univariate and multivariate logistic regression analyses result

Univariate analysis showed that the absence or presence of hypertriglyceridemia, tumor location, lesion size, macroscopic type, invasion depth, differentiation, absence or presence of LVI, and perineural invasion were significantly associated with LNM. Eight significant risk factors were included in multivariate logistic regression analysis (Table 5). Based on multivariate logistic regression analysis, hypertriglyceridemia (*P* = .021), tumors located in the lower thoracic esophagus (*P* = .012), lesion size > 20 mm (*P* = .027), submucosal invasion (*P* = .012), and LVI (*P* = .030) were independent risk factors for LNM. The linear analysis showed that their VIF was <5, and there was no multicollinearity between the 5 factors.

**Table 5:** Multivariate logistic regression analysis of lymph node metastasis in superficial esophageal cancer.

Factor	B	SE	Wald	*P* value	OR	95%CI
Hypertriglyceridemia						
Absence					Reference	
Presence	2.321	0.999	5.398	**.020**	10.186	1.438–72.168
Location						
Upper + Middle					Reference	
Lower	2.878	1.156	6.201	**.013**	17.780	1.846–171.276
Lesion size						
≤20mm					Reference	
>20mm	1.953	0.882	4.899	**.027**	7.048	1.251–39.720
Macroscopic type						
I(Protruded)					Reference	
II(Flat)	-0.392	1.043	0.141	.707	0.676	0.087–5.220
III(Depressed)	-2.181	1.510	2.087	.149	0.113	0.006–2.178
Invasion depth				**.012**		
Mucosa					Reference	
SM1	3.216	1.337	5.785	.016	24.929	1.814–342.644
SM2 + SM3	1.038	1.413	0.539	.463	2.824	0.177–45.075
Differentiation				.562		
Well differentiated					Reference	
Moderately differentiated	1.700	1.741	0.953	.329	5.471	0.180–165.900
Poorly differentiated	1.120	1.759	0.406	.524	3.065	0.098–96.288
LVI						
Absence					Reference	
Presence	2.686	1.239	4.700	**.030**	14.674	1.294–166.400
Perineural invasion						
Absence					Reference	
Presence	-0.597	1.486	0.161	.688	0.551	0.030–10.140

CI *=* Confidence interval, LNM *=* Lymph node metastasis, LVI *=* Lymphovascular invasion, OR *=* Odds ratio, SE *=* standard error, SM1 *=* upper third of submucosa layer, SM2 *=* middle third of submucosa layer, SM3 *=* lower third of submucosa layer.

### 3.5. Univariate analysis of hypertriglyceridemia

Unlike previous studies, we included the presence or absence of hyperlipidemia in this study and found that hyperlipidemia was a risk factor for LNM in SESCC patients. However, whether hyperlipidemia indirectly affects LNM by influencing the depth of invasion or other factors needs to be further studied. Therefore, we further analyzed hyperlipidemia by chi-square test (Table 6). As a result, the presence or absence of hyperlipidemia was only related to the presence or absence of perineural invasion, not to tumor location, lesion size, macroscopic type, invasion depth, differentiation, and absence or presence of LVI.

**Table 6:** Univariate analysis of hypertriglyceridemia

	Hypertriglyceridemia		*P* value
	Absence	Presence	
Location			.423
Upper + Middle	45 (78.9)	12 (21.1)	
Lower	48 (72.7)	18 (27.3)	
Lesion size			.731
≤20mm	56 (76.7)	17 (23.3)	
>20mm	37 (74.0)	13 (26.0)	
Macroscopic type			.495
Flat	31 (79.5)	8 (20.5)	
No flat	62 (73.8)	22 (26.2)	
Invasion depth			.664
Mucosa	39 (78.0)	11 (22.0)	
SM1	28 (77.8)	8 (22.2)	
SM2 + SM3	26 (70.3)	11 (29.7)	
Differentiation			.193
Well differentiated	24 (82.8)	5 (17.2)	
Moderately differentiated	52 (77.6)	15 (22.4)	
Poorly differentiated	17 (63.0)	10 (37.0)	
LVI			.237
Absence	84 (77.8)	24 (22.2)	
Presence	9 (60.0)	6 (40.0)	
Perineural invasion			**.011**
Absence	91 (78.4)	25 (21.6)	
Presence	2 (28.6)	5 (71.4)	

LVI = Lymphovascular invasion, SM1 = upper third of submucosa layer, SM2 = middle third of submucosa layer, SM3 = lower third of submucosa layer.

### 3.6. Development of an LNM-predicting nomogram

**Figure 8.**
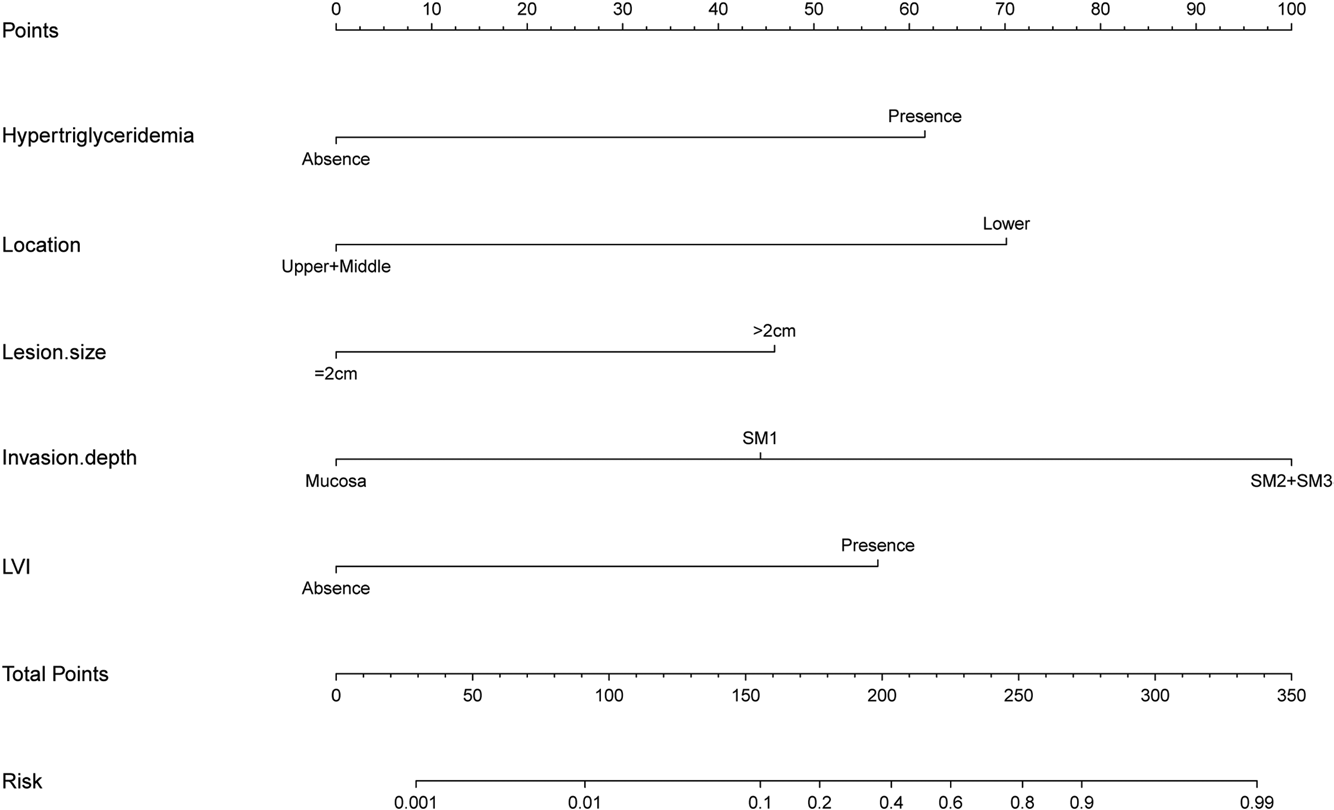
Nomogram for predicting lymph node metastasis in patients with superficial esophageal squamous cell carcinoma. There are 8 rows in the nomogram. The variables are presented in rows 2 to 6. Locate the patient characteristics on each variable axis and draw a vertical line on the points axis (top) to assign a point value to the variable. Sum the total points and draw a vertical line from the total points axis to get the probability of lymph node metastasis.

**Figure 9.**
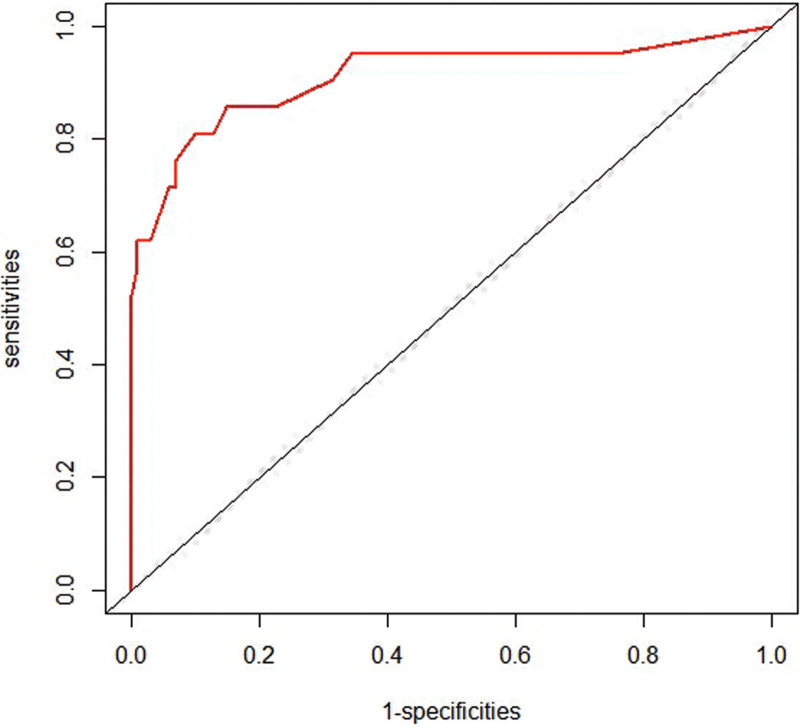
Receiver operating characteristic (ROC) curve of the model. The area under the ROC curve was 0.937 (95% confidence intervals:0.874–0.999).

**Figure 10.**
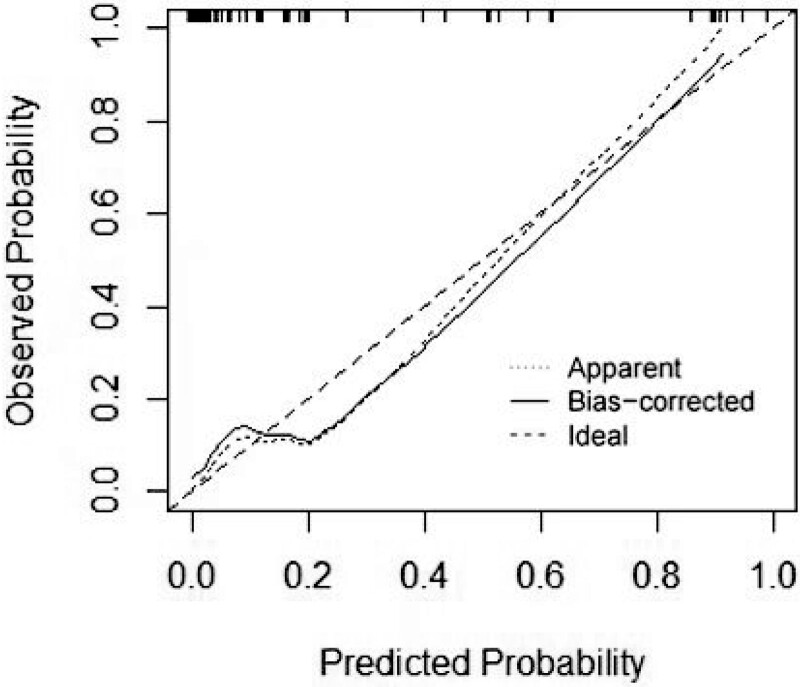
Calibration plots of the nomogram.

A nomogram that was established based on the risk factors was confirmed using multivariate logistic regression analysis (Fig. 8). Using this nomogram, we can predict the probability of LNM by adding the scores for each factor and projecting the total score to the bottom scale, which can guide clinicians in choosing optimal treatment. This nomogram showed good discrimination with an the area under the receiver operating characteristic curve and concordance index of 0.937 (95%CI:0.874–1) (Fig. 9). The Hosmer-Lemeshow test revealed that the χ^2^ was 1.468 (*P* = .480). The Brier score was 0.056 and the calibration plot for bootstrap resampling validation showed good agreement between the observed and predicted results (Fig. 10). After bootstrapping, AUC was 0.909, indicating a good model fit.

## 4. Discussion

In previous studies have shown that patients with SESCC have a better prognosis than those with advanced esophageal cancer,^[18,19]^ but their survival rate decreased significantly once LNM occurred.^[20,21]^ The standard treatment for patients with SESCC has been radical resection with lymph node dissection, but it has the disadvantages of being invasive and affecting the subsequent quality of life. According to the JES guidelines,^[11]^ ER may be considered for patients with SESCC with a low risk of LNM after careful preoperative evaluation. However, it is difficult to accurately predict whether LNM will occur, based on existing technology. In this study, we researched the most factors included clinical characteristics, endoscopic features, and pathological characteristics that may be associated with LNM, analyzed independent risk factors for LNM, and plotted a nomogram based on the results. Finally, the absence or presence of hypertriglyceridemia, tumor location, lesion size, invasion depth, and absence or presence of LVI were included in the model. The model was validated internally and showed good calibration and discrimination. The nomogram can be used clinically to assess the risk of LNM and select appropriate treatment modalities.

In this study, we enrolled patients with hypertriglyceridemia and hypercholesterolemia, which had never been seen in previous study. Univariate and multivariate analyses revealed that the presence or absence of hypertriglyceridemia was an independent risk factor of LNM. LNM was significantly more frequent in patients with hypertriglyceridemia. TG is synthesized from lipids, which are derived from food in the intestinal epithelium and is absorbed from the intestinal lymphatics into the celiac complex by degrading lipoprotein lipase expressed on the endothelial cells^[22,23]^ and converted into lysophosphatidic acid (LPA), which is a powerful stimulator of a wide range of cellular functions involved in tumor and metastasis development.^[24,25]^ Thus, lymphatic endothelial cells in the esophagus of patients with hypertriglyceridemia are frequently affected by high concentrations of TG produced by food intake and are rich in LPA, making them more susceptible to LNM.^[26]^ Furthermore, Zheng et al found that hyperlipidemia is an independent risk factor for postoperative recurrence of esophageal cancer. An earlier postoperative recurrence time is related to higher lipid level.^[27]^

Our study found that the tumor location in SESCC was closely related to LNM. Tumors which were located in the lower thoracic esophagus were more prone to LNM than those located in the upper-middle thoracic esophagus, and this is consistent with the findings of Wu et al.^[28,29]^ However, some researches reached contrary conclusions, arguing that LNM has nothing to do with tumor location.^[30,31]^ That current guidelines do not specify the location of tumors that can be resected endoscopically, requires further research.

Our study also found that lesion size > 20 mm, submucosal invasion, and LVI were independent risk factors for LNM, which is consistent with the findings of Ruan et al,^[32]^ Jiang et al^[33]^ and Xu et al.^[34]^ Taking the tumor size ≤ 20 mm as a reference, the odds ratio of tumor size > 20 mm was 6.956 (95% CI 1.244–38.913) for predicting LNM, indicating that the lesion size is an important risk factor for LNM. The reason for the large 95% confidence interval may be the small size of the data; however, it still showed that there was a strong relevance between lesion size and LNM. As for invasion depth, the LNM rates in the mucosa, SM1, and SM2 + SM3 were 2.0%, 8.3%, and 45.9%, respectively. Previous publications showed that tumors in the EP to LPM layers had a low incidence of LNM (0–5.6 %). In contrast, incidence of LNM in the SM2 and SM3 layers ranged from 18% to 67%,^[35]^ which are consistent with those of our study. To further validate the guidelines and select the most appropriate treatment, we stratified the depth of invasion in more detail into EP + LPM, MM + SM1, and SM2 + SM3.The LNM rates in these 3 groups were 0%, 6.2%, and 45.9%, respectively. Zhou et al showed LNM rates in patients with SESCC with EP, LPM, MM, SM1, SM2, and SM3 lesions as 0%, 0%, 9.78%, 16.92%, 13.73%, and 35.29%, respectively.^[10]^ The low incidence of LNM with tumor infiltration into MM + SM1 in our study may be affected by other factors, such as tumor size, tumor location, presence of vascular invasion, and nerve invasion. This shows that the absolute indication in the guidelines is also applicable to Chinese patients, and for patients who meet the absolute indications, ER can be considered first. Patients meeting the relative indication still have the risk of LNM, although the risk is low. Now, we can apply the nomogram we established to further comprehensively evaluate the risk of LNM in order to choose the most suitable treatment. However, when the tumor infiltrates SM2 or SM3, the possibility of LNM is greatly increased; thus, radical surgery should be considered first. Several clinical and pathological studies have confirmed a close relationship between LVI and LNM,^[28,30–32,35]^ and our study confirmed this. LNM occurred in only 10.2% of patients without LVI, but once LVI occurred, the probability of LNM was greatly increased to 66.7%. Therefore, when a patient with SECSC has LVI, clinicians should choose ER with caution and evaluate the need for additional surgery or other treatments after ER.

In contrast to other studies,^[31,36]^ differentiation could not strongly predict LNM in the multivariate logistic regression analysis in our study. This may be due to confounding by other predictive risk factors, including invasion depth and tumor size. Additionally, in a study by Semenkovich et al, histology was found to be an independent risk factor for LNM.^[37]^ Jia et al found that the macroscopic type was an independent risk factor.^[38]^ However, in our study, the macroscopic type was significantly different in univariate analysis, but not in multivariate analysis, which may be due to an influence by other factors.

There are some limitations in this study. First, the sample size was small, and there may be statistical bias related to the small sample size. Second, this retrospective study was implemented in a single institution, and we only performed internal validation. Therefore, a multicenter prospective study, well designed with external validation, is required. Third, to facilitate data analysis, we did not finely divide the invasion depth into 6 layers (at mucosal level, including EP, LPM, and MM; in the submucosa, including SM1, SM2, and SM3) in the nomogram. Finally, this nomogram was based on Chinese patients only and may not be applicable to Western populations.

## 5. Conclusion

In conclusion, we identified the risk factors for LNM in patients with SESCC using univariate and multivariate analyses, including hypertriglyceridemia, tumors located in the lower thoracic esophagus, lesion size > 20 mm, submucosal invasion, and LVI. Hypertriglyceridemia is an innovation factor that has rarely been researched before. A nomogram was constructed using the 5 independent risk factors. This model can help clinicians assess the risk of LNM in patients with SESCC, for optimal treatment selection.

## Acknowledgments

The authors would like to thank Professor Zibin Tian for modifying the content of the article and thank Editage for English language editing.

## Author contributions

**Data curation:** Hanqing Li, Xuan Zhou.

**Methodology:** Zitong Xu, Xiaoyu Li, Yuning Chu, Minghan Ren.

**Writing – original draft:** Jing Wang.

**Writing – review & editing:** Xiangji Liu, Tao Mao, Zibin Tian.
